# *Candida auris:* epidemiological situation, laboratory capacity and preparedness in European Union and European Economic Area countries, 2013 to 2017

**DOI:** 10.2807/1560-7917.ES.2018.23.13.18-00136

**Published:** 2018-03-29

**Authors:** Anke Kohlenberg, Marc J Struelens, Dominique L Monnet, Diamantis Plachouras, The Candida auris survey collaborative group

**Affiliations:** 1 European Centre for Disease Prevention and Control (ECDC), Stockholm, Sweden; 2 The members of the group are listed at the end of the article

**Keywords:** Candida auris, multidrug-resistance, outbreaks, surveillance, preparedness and response, laboratory capacity, mycology

## Abstract

During 2013–2017, 620 cases of *Candida auris* were reported in the European Union/European Economic Area – 466 (75.2%) colonisations, 110 (17.7%) bloodstream infections, 40 (6.5%) other infections and four cases (0.6%) of unknown colonisation/infection status – the majority from four large outbreaks. Survey results showed that several countries lacked laboratory capacity and/or information on the occurrence of cases at national level. To prevent further spread, adequate laboratory capacity and infection control preparedness is required in Europe.

Between 2015 and 2016, outbreaks of *Candida auris* occurred in European countries and these triggered a rapid risk assessment from the European Centre for Disease Prevention and Control (ECDC) [1]. To follow-up if these outbreaks had been controlled and determine the current situation regarding *C. auris* in Europe, an online survey was conducted in early 2018.

*C. auris* is an emerging fungus that is causing difficult-to-control outbreaks of invasive healthcare-associated infections. Since the first report of *C. auris* in 2009 [2], cases have been reported worldwide. Identification of *C. auris* requires specialised laboratory methodology as traditional identification methods may lead to misidentification [3,4]. In addition, *C. auris* has been associated with resistance to multiple antifungal classes [5] and difficulties related to the interpretation of antifungal susceptibility results [6]. The combination of these characteristics, i.e. propensity to cause nosocomial outbreaks, multi-drug resistance, ability to cause severe disease and difficulties with laboratory detection, render *C. auris* a public health threat for European healthcare facilities.

## Survey on reported cases and laboratory capacity in Europe

In December 2016, 85 *C. auris* cases reported by four European Union/European Economic Area (EU/EEA) countries were described in a rapid risk assessment issued by ECDC [1]. To determine the epidemiological situation at the start of 2018, and to assess laboratory capacity for *C. auris* detection in the EU/EEA, we invited the National Focal Points for collaboration with ECDC for healthcare-associated infections or their deputies, to complete a survey in January 2018. This survey included 12 questions on the aggregated number of *C. auris* cases and outbreaks reported per year in the period from 2013 to 2017, the national capacity for laboratory identification and antifungal susceptibility testing of *C. auris* as well as the public health actions taken in response to alerts issued in 2016 by the ECDC, the United States (US) Centers for Disease Control and Prevention (CDC) and Public Health England (PHE) [1,7,8].

A case of *C. auris* was defined as a patient in whom *C. auris* was detected and this definition included patients with invasive *C. auris* infection as well as colonised patients without invasive disease.

## Reported cases and outbreaks

**Figure 1:**
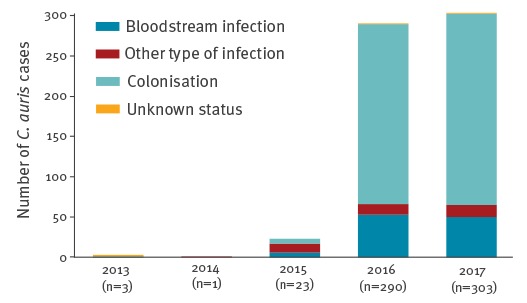
Number of reported *Candida auris* cases by year and infection or colonisation, European Union and European Economic Area countries, 2013–2017 (n = 620)^a^

^a^ One additional case detected in Austria in January 2018 is not included in the figure.

We received replies to the survey from 29 of 30 EU/EEA countries with separate replies from the United Kingdom (UK) for England and Scotland. From 2013 to 2017, a total of 620 *C. auris* cases were reported from six countries (Figure 1).

**Figure 2:**
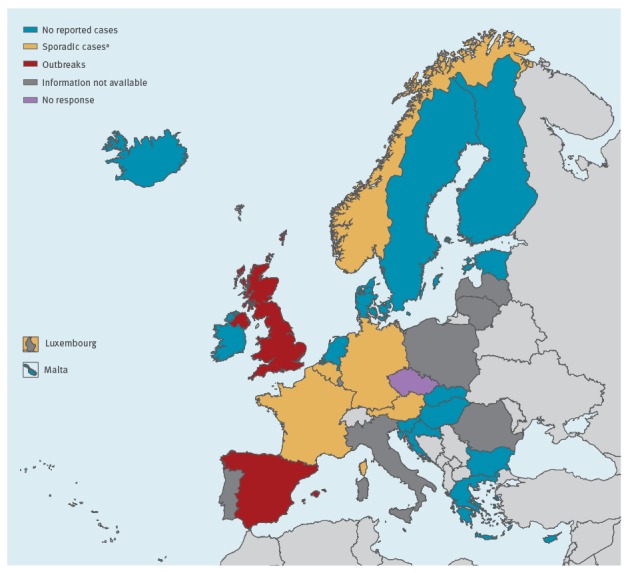
Geographic distribution of *Candida auris* cases reported in European Union / European Economic Area countries, 2013–2017 (n = 620)^a^

^a^ The map includes one additional case detected in Austria in January 2018 that is not included in the total for the period 2013–2017.

Cases were reported from Spain (n = 388), the UK (n = 221), Germany (n = 7), France (n = 2), Belgium (n = 1) and Norway (n = 1) in the period from 2013 to 2017. Austria detected one case in January 2018. The majority of cases were reported as colonisations (n = 466; 75.2%), while a bloodstream or other type of infection was reported in 150 cases (24.2%). For four cases (0.6%) the colonisation/infection status was unknown. The annual number of cases and information on the infection or colonisation status and the type of infection (bloodstream or other type) are shown in Table 1. No *C. auris* colonisation or invasive infection had been detected in 15 countries and in seven countries the National Focal Points did not have information on *C. auris* cases available at the national level (Figure 2).

**Table 1:** Number of *Candida auris* cases detected in the European Union/European Economic Area, 2013–2017 (n = 620)^a^

Year	*Candida auris* bloodstream infection		Other type of *C. auris* infection		*C. auris* colonisation		Cases of unknown infection/ colonisation status		Total
	**n**	**%**	**n**	**%**	**n**	**%**	**n**	**%**	**n**
**2013**	1	33.3	0	0.0	0	0.0	2	66.7	3
**2014**	0	0.0	1	100.0	0	0.0	0	0.0	1
**2015**	6	26.1	11	47.8	6	26.1	0	0.0	23
**2016**	53	18.3	13	4.5	223	76.9	1	0.3	290
**2017**	50	16.5	15	5.0	237	78.2	1	0.3	303
**2013–2017**	110	17.7	40	6.5	466	75.2	4	0.6	620

All percentages are row percentages.

^a^ One additional case detected in Austria in January 2018 is not included in the table.

Sporadic cases include one case for Austria, one case for Belgium, two cases for France, seven cases for Germany and one case for Norway.

Two countries had experienced four nosocomial outbreaks of *C. auris* affecting 573 patients in total. The number of cases per outbreak ranged from 39 to 382 according to national reporting. Inter-facility transmission occurred in the four outbreaks and one outbreak lasted nearly 2 years. Three outbreaks were controlled whereas one outbreak was still ongoing as at January 2018. Measures to control these outbreaks included contact precautions, single room isolation, cohorting, contact screening and enhanced environmental disinfection.

## Laboratory capability

Laboratory capability to detect and identify *C. auris* was available in 21 of the 29 responding countries, either by formally designated mycology reference laboratories (n = 12 countries) or by laboratories with a reference function (n = 9 countries) (Table 2). Methods used for identification of *C. auris* were proteomic analysis with Matrix-Assisted Laser Desorption Ionization-Time of Flight Mass Spectrometry (MALDI-TOF) (n = 17 laboratories), sequencing of genetic loci, including of D1/D2 locus (n = 9 laboratories), and sequencing of internal transcribed spacer (ITS) domains of the rRNA (n=6 laboratories). Antifungal susceptibility testing for azoles, amphotericin B and echinocandins was available in all reference laboratories or laboratories with reference function except one (Table 2).

**Table 2:** National laboratory capacity for *Candida auris* identification and testing and public health measures taken in response to *C. auris* alerts in the European Union/European Economic Area, as at January 2018

Country	MRL/ laboratory with reference function	Antifungal susceptibility testing^a^ at reference laboratory	Clinical alert	Laboratory alert	Offer of reference testing to hospital laboratories	Development of guidance for laboratory testing	Development of guidance for clinical management	Development of guidance for infection control	Retrospective surveillance	Prospective surveillance
Austria	**Y**	**Y**	**Y**	**Y**	**Y**	**Y**	**Y**	N	**Y**	N
Belgium	**Y**	**Y**	N	N	N	N	N	N	N	N
Bulgaria	**Y**	**Y**	N	N	**Y**	N	N	N	N	N
Croatia	**Y**	**Y**	N	**Y**	N	**Y**	N	**Y**	N	N
Cyprus	N	N	N	N	N	N	N	N	N	N
Denmark	**Y**	**Y**	**Y**	**Y**	**Y**	N	N	N	**Y**	**Y**
Estonia	N	N	**Y**	**Y**	**Y**	N	N	N	N	N
Finland	**Y**	**Y**	**Y**	N	N	N	N	N	N	N
France	**Y**	**Y**	N	Y	**Y**	N	N	N	**Y**	**Y**
Germany	**Y**	**Y**	N	**Y**	**Y**	**Y**	N	N	N	**Y**
Greece	**Y**	**Y**	**Y**	**Y**	**Y**	N	N	N	**Y**	N
Hungary	**Y**	**Y**	N	N	N	N	N	N	N	N
Iceland	**Y**	**Y**	N	**Y**	**Y**	N	N	N	N	**Y**
Ireland	N	N	**Y**	**Y**	N	N	N	N	N	**Y**
Italy	N	N	N	N	N	N	N	N	N	N
Latvia	N	N	N	N	N	N	N	N	N	N
Lithuania	**Y**	**Y**	**Y**	N	N	N	N	N	N	N
Luxembourg	**Y**	N	N	N	N	N	N	N	N	N
Malta	**Y**	**Y**	N	**Y**	N	**Y**	N	N	N	N
The Netherlands	**Y**	**Y**	N	**Y**	N	N	N	N	**Y**	N
Norway	**Y**	**Y**	N	**Y**	N	**Y**	**Y**	**Y**	N	N
Poland	N	N	N	N	N	N	N	N	N	N
Portugal	**Y**	**Y**	N	**Y**	N	N	N	N	N	N
Romania	N	N	N	N	N	N	N	N	N	N
Slovakia	N	N	N	N	N	N	N	N	N	N
Slovenia	**Y**	**Y**	N	**Y**	**Y**	N	N	N	N	**Y**
Spain	**Y**	**Y**	**Y**	**Y**	**Y**	N	N	N	N	N
Sweden	**Y**	**Y**	N	**Y**	**Y**	N	N	N	N	N
UK-England	**Y**	**Y**	**Y**	**Y**	**Y**	**Y**	**Y**	**Y**	**Y**	**Y**
UK-Scotland	**NA^b^**	**NA^b^**	**Y**	**Y**	**Y**	**Y**	**Y**	**Y**	**Y**	**Y**

NA: not applicable; MRL: mycology reference laboratory**;** UK: United Kingdom.

^a^ Antifungal susceptibility testing includes susceptibility testing for azoles, amphotericin B and echinocandins.

**^b^** UK-Scotland is able to use the reference laboratory facilities in Bristol UK-England as required. The need for a Scotland specific reference laboratory is under review.

In the table, a Y and blue shading signifies ‘yes, in place’ (columns 2–3) or ‘yes, performed’ (columns 4–11) and N signifies ‘not in place’ (columns 2–3) or ‘not performed’ (columns 4–11).

Public health measures for preparedness or response to *C. auris* had been taken in 20 countries. The most frequent measures taken were dissemination of laboratory (n = 18 countries) or clinical alerts (n = 10 countries,) and offers for reference identification and antifungal susceptibility testing to hospital laboratories (n = 13 countries). Preparation of guidance for laboratory testing (n = 7 countries), clinical management (n = 4 countries) or infection control (n = 4 countries) was undertaken less frequently, and retrospective or prospective surveillance was in place in only few countries (n = 8 and 7 countries, respectively) (Table 2).

## Discussion

Our results show an increasing number of *C. auris* colonisations and invasive infections in the EU/EEA since 2013. Large and prolonged nosocomial outbreaks of *C. auris* have occurred in two countries between 2015 and 2017, confirming the potential of *C. auris* as a healthcare-associated pathogen and the difficulties encountered in controlling its spread. Outbreaks of *C. auris* have also been described from four other continents from countries such as Venezuela [9], South Africa [10], the US [11] and India [12] showing that, within a few years, *C. auris* has become a global public health issue and that further outbreaks can be expected.

The increasing number of sporadic cases, mostly invasive infections, compared with the ECDC rapid risk assessment in 2016 [1] confirms that introduction of *C. auris* into hospitals in Europe is occurring repeatedly, each time with the potential risk for further transmission and healthcare-associated outbreaks. The reason why some cases of *C. auris* have caused large outbreaks while other cases were sporadic with no apparent further transmission remains unclear. However, reports of sporadic cases might represent a ‘tip of the iceberg’ phenomenon, as only few isolates might reach mycology reference laboratories and no information is available whether contact screening surrounding these sporadic cases was performed to exclude further transmission.

Early detection of *C. auris* is necessary for preventing further colonisations, invasive infections, and outbreaks. With the increasing number of *C. auris* cases in the EU/EEA, it is of concern that some countries still lack national laboratory reference capacity for mycology or have no information on *C. auris* cases available at national public health level. Due to the lack of laboratory capability for routine detection and surveillance, recognition of *C. auris* introduction into a healthcare facility might be delayed until spread has already occurred. Mycology reference laboratory capacity is all the more important due to the increasing use of immunosuppressive therapy, antimicrobial and antifungal treatment or prophylaxis that increase the risk for fungal infection or the risk for antifungal drug resistance [13,14]. The emergence of *C. auris* with the propensity to spread, cause invasive infections and survive in the environment, further highlights the need for adequate mycology reference capacity.

Mycology reference laboratory capacity can only contribute to *C. auris* control if clinicians and hospital laboratories are aware of this threat and react in a timely way to an increase in severe *Candida* spp. infections or detection of an isolate of *C. auris*. This also requires an increased effort for more extensive speciation of *Candida* spp. isolates from bloodstream and other invasive infections and, under certain circumstances, for example if *C. auris* has already been detected in a healthcare facility, also of isolates from other non-sterile body sites [15,16]. The occurrence of a single case of *C. auris* in a hospital requires an adequate response to prevent further spread [17]. However, not all of the surveyed countries have so far issued clinical or laboratory alerts to increase awareness at hospital level.

In conclusion, *C. auris* is detected with increasing frequency and large outbreaks have occurred in Europe since 2013. To mitigate the risk from the introduction of *C. auris* and to prevent and control its further spread, adequate laboratory capacity, surveillance, and infection control preparedness is required in all EU/EEA countries.

## Notes

**The *Candida auris* survey collaborative group:** Austria: Petra Apfalter, Cornelia Lass-Flörl, Robert Muchl, Elisabeth Presterl, Birgit Willinger; Belgium: Boudewijn Catry; Bulgaria: Ivva Philipova; Crotia: Ana Budimir; Cyprus: Linos Hadjihannas; Denmark: Maiken Cavling Arendrup; Estonia: Pille Märtin; Finland: Jari Jalava; France: Anne Berger-Carbonne, Francoise Dromer; Germany: Oliver Kurzai; Greece: Georgia Vrioni, Flora Kontopidou; Hungary: Katalin Kiss, Andrea Kurcz; Iceland: Lena Rós Ásmundsdóttir; Ireland: Karen Burns; Italy: Maria Luisa Moro; Latvia: Elina Dimiņa; Lithuania: Rolanda Valintėlienė; Luxembourg: Monique Perrin; Malta: Christopher Barbara; Netherlands: Sabine de Greeff; Norway: Oliver Kacelnik; Poland: Aleksander Deptuła; Portugal: Maria do Rosário Rodrigues; Romania: Roxana Serban; Slovakia: Eva Schréterová, Maria Stefkovicova; Slovenia: Mojca Serdt; Spain: Ana Alastruey-Izquierdo, Javier Pemán; Sweden: Sara Byfors, Erja Chryssanthou; UK-England: Andy Borman, Colin Brown, Rebecca Guy, Elizabeth Johnson; UK-Scotland: Jacqui Reilly.

## References

[1] European Centre for Disease Prevention and Control (ECDC). Candida auris in healthcare settings -Europe. Stockholm: ECDC; 2016. Available from: https://ecdc.europa.eu/sites/portal/files/media/en/publications/Publications/Candida-in-healthcare-settings_19-Dec-2016.pdf

[2] Satoh K Makimura K Hasumi Y Nishiyama Y Uchida K Yamaguchi H Candida auris sp. nov., a novel ascomycetous yeast isolated from the external ear canal of an inpatient in a Japanese hospital. Microbiol Immunol. 2009;53(1):41-4. 10.1111/j.1348-0421.2008.00083.x 19161556

[3] Jeffery-Smith A Taori SK Schelenz S Jeffery K Johnson EM Borman A Candida auris: a Review of the Literature. Clin Microbiol Rev. 2017;31(1):e00029-17. 10.1128/CMR.00029-17 29142078 PMC5740969

[4] Spivak ES Hanson KE Candida auris: an Emerging Fungal Pathogen. J Clin Microbiol. 2018;56(2):e01588-17. 29167291 10.1128/JCM.01588-17 PMC5786713

[5] Lamoth F Kontoyiannis DP The Candida auris Alert: Facts and Perspectives. J Infect Dis. 2018;217(4):516-20. 10.1093/infdis/jix597 29390110

[6] Arendrup MC Prakash A Meletiadis J Sharma C Chowdhary A Comparison of EUCAST and CLSI Reference Microdilution MICs of Eight Antifungal Compounds for Candida auris and Associated Tentative Epidemiological Cutoff Values. Antimicrob Agents Chemother. 2017;61(6):e00485-17. 10.1128/AAC.00485-17 28416539 PMC5444165

[7] Centers for Disease Control and Prevention (CDC). Clinical alert to U.S. healthcare facilities: global emergence of invasive infections caused by the multidrug-resistant yeast Candida auris. Atlanta: CDC; 2016. Available from: https://www.cdc.gov/fungal/diseases/candidiasis/candida-auris-alert.html

[8] Public Health England (PHE). Candida auris identified in England. London: PHE; 2016. Available from: https://www.gov.uk/government/publications/candida-auris-emergence-in-england/candida-auris-identified-in-england

[9] Calvo B Melo AS Perozo-Mena A Hernandez M Francisco EC Hagen F First report of Candida auris in America: Clinical and microbiological aspects of 18 episodes of candidemia. J Infect. 2016;73(4):369-74. 10.1016/j.jinf.2016.07.008 27452195

[10] National Institute for Communicable Diseases. South Africa. Candida auris outbreak in the neonatal unit of a Johannesburg public-sector hospital. Johannesburg: NICD; 2017. Available from: http://www.nicd.ac.za/wp-content/uploads/2017/09/NICD-Communicable-Diseases-Communique_September2017_final.pdf

[11] Tsay S Welsh RM Adams EH Chow NA Gade L Berkow EL Notes from the Field: Ongoing Transmission of Candida auris in Health Care Facilities - United States, June 2016-May 2017. MMWR Morb Mortal Wkly Rep. 2017;66(19):514-5. 10.15585/mmwr.mm6619a7 28520710 PMC5657645

[12] Chowdhary A Sharma C Duggal S Agarwal K Prakash A Singh PK New clonal strain of Candida auris, Delhi, India. Emerg Infect Dis. 2013;19(10):1670-3. 10.3201/eid1910.130393 24048006 PMC3810747

[13] Wiederhold NP Antifungal resistance: current trends and future strategies to combat. Infect Drug Resist. 2017;10:249-59. 10.2147/IDR.S124918 28919789 PMC5587015

[14] Kullberg BJ Arendrup MC Invasive Candidiasis. N Engl J Med. 2015;373(15):1445-56. 10.1056/NEJMra1315399 26444731

[15] Centers for Disease Control and Prevention (CDC). Candida auris Clinical Update - September 2017 Atlanta: CDC; 2017. Available from: https://www.cdc.gov/fungal/diseases/candidiasis/c-auris-alert-09-17.html

[16] Lockhart SR Jackson BR Vallabhaneni S Ostrosky-Zeichner L Pappas PG Chiller T Thinking beyond the Common Candida Species: Need for Species-Level Identification of Candida Due to the Emergence of Multidrug-Resistant Candida auris. J Clin Microbiol. 2017;55(12):3324-7. 10.1128/JCM.01355-17 28904185 PMC5703798

[17] Tsay S Kallen A Jackson BR Chiller TM Vallabhaneni S Approach to the Investigation and Management of Patients With Candida auris, an Emerging Multidrug-Resistant Yeast. Clin Infect Dis. 2018;66(2):306-11. 10.1093/cid/cix744 29020224 PMC5798232

